# Perfusion MRI Derived Indices of Microvascular Shunting and Flow Control Correlate with Tumor Grade and Outcome in Patients with Cerebral Glioma

**DOI:** 10.1371/journal.pone.0123044

**Published:** 2015-04-13

**Authors:** Anna Tietze, Kim Mouridsen, Yasmin Lassen-Ramshad, Leif Østergaard

**Affiliations:** 1 Department of Neuroradiology, Aarhus University Hospital, Aarhus, Denmark; 2 Center of Functionally Integrative Neuroscience, Aarhus University, Aarhus, Denmark; 3 Department of Oncology, Aarhus University Hospital, Aarhus, Denmark; University of Pécs Medical School, HUNGARY

## Abstract

**Objectives:**

Deficient microvascular blood flow control is thought to cause tumor hypoxia and increase resistance to therapy. In glioma patients, we tested whether perfusion-weighted MRI (PWI) based indices of microvascular flow control provide more information on tumor grade and patient outcome than does the established PWI angiogenesis marker, cerebral blood volume (CBV).

**Material and Methods:**

Seventy-two glioma patients (sixty high-grade, twelve low-grade gliomas) were included. Capillary transit time heterogeneity (CTH) and the coefficient of variation (COV), its ratio to blood mean transit time, provide indices of microvascular flow control and the extent to which oxygen can be extracted by tumor tissue. The ability of these parameters and CBV to differentiate tumor grade were assessed by receiver operating characteristic curves and logistic regression. Their ability to predict time to progression and overall survival was examined by the Cox proportional-hazards regression model, and by survival curves using log-rank tests.

**Results:**

The best prediction of grade (AUC = 0.876; p < 0.05) was achieved by combining knowledge of CBV and CTH in the enhancing tumor and peri-focal edema, and patients with glioblastoma multiforme were identified best by CTH (AUC = 0.763; p<0.001). CTH outperformed CBV and COV in predicting time to progression and survival in all gliomas and in a subgroup consisting of only high-grade gliomas.

**Conclusion:**

Our study confirms the importance of microvascular flow control in tumor growth by demonstrating that determining CTH improves tumor grading and outcome prediction in glioma patients compared to CBV alone.

## Introduction

Hypoxic tumors tend to grow and metastasize faster than well-oxygenated tumors, and to be more resistant to radio- and chemotherapy [1]. In high-grade gliomas, the extent of tumor hypoxia is correlated with time to progression and overall survival [2]. Tumors secure their supply of oxygen and other nutrients by stimulating tissue angiogenesis, and the extent of vessel formation is closely related to tumor development and is an indirect marker of high malignancy and aggressiveness. Accordingly, cerebral blood volume (CBV) maps determined by perfusion-weighted MRI (PWI), correlate with the vascular density in cerebral gliomas and provide important prognostic information [3–7]. The extent to which newly formed tumor vessels support tumor oxygenation remains, however, less well understood. It has been hypothesized that the excessive growth of immature, tortuous, and hyperpermeable microvessels impair the delivery of both nutrients and therapeutics to tumor tissue [8,9]. By introducing functional shunts through the tumor microvasculature, dysfunctional tumor vessels are in fact thought to contribute to tumor hypoxia and tumor necrosis [10].

To understand the role of the microvasculature in the extraction of oxygen and other diffusible substances in tissue, we recently extended the so-called flow-diffusion equation to take the capillary transit time heterogeneity (CTH) into account [11]. The ‘traditional’ flow-diffusion equation [12] states that the extraction of freely diffusible substances in tissue depends on tissue blood flow, capillary surface area, and capillary permeability to the substance. We noted, however, that this equation requires capillaries to be homogeneously perfused, an assumption that is unlikely to apply to tumor vasculature [13]. In tumors, CTH can be elevated by abnormal capillary bed topology, shunts, microthrombosis, pericyte loss, edema pressure, etc. [10]. The extended flow-diffusion equation permitted us to quantify how elevated CTH limits the availability of oxygen, and favors excessive glucose break-down (aerobic glycolysis) in tumors with a heterogeneously perfused tissue microcirculation [13]. We have now developed a method that allows CTH to be determined by dynamic susceptibility contrast (DSC) MRI data of the type acquired during routine clinical examinations [14].

This study tests the predicted relation between CTH and tumor microenvironment by examining whether CTH differs among high-grade (HGG) and low-grade gliomas (LGG) and whether knowledge of this physiological parameter translates into a pre-surgical predictor of time to progression (TTP) and overall survival (OS). We compare CTH with CBV, the traditional MRI marker for angiogenesis in tumors, in order to ascertain whether information of capillary flow patterns provides diagnostic or prognostic information in addition to that provided by the extent of neo-angiogenesis. For different tumor types and grades, we describe CTH in the enhancing tumor, in peri-tumoral edema, and in normal appearing white matter and discuss their relation to tumor pathology and edema.

## Materials and Methods

### Patients

The Danish Committee on Health Research Ethics (local committee: Central Denmark Region) specifically approved this retrospective study. All patients gave written informed consent. All patient data were anonymized prior to analysis. 72 patients (24 females) were consecutively enrolled between November 2010 and October 2012. The diagnosis of glioma (sixty HGGs, twelve LGGs) was obtained by biopsy, sub-total or total resection, using post-contrast T1-weighted or, in case of non-enhancing lesions, T2FLAIR images as surgical guidance. In total, 41 patients with glioblastoma multiforme (GBM), 14 with astrocytoma grade 3 (AC3), 5 with oligodendroglioma grade 3 (ODG3), 7 with astrocytoma grade 2 (AC2), and 3 with oligodendroglioma grade 2 (ODG2) were included. One tumor was classified as an astrocytoma grade 2 with a minor oligodendroglial component, subsequently included into the grade 2 astrocytoma group. Patients with GBM were subsequently treated with the Stupp regimen [15] consisting of radio-chemotherapy (temozolomide), grade 3 tumors with radiotherapy, and LGGs were surgically treated and clinically observed. In case of disease progression, some GBMs received anti-angiogenic treatment (bevacizumab combined with irinotecan). In case of recurrence, grade 3 tumors were usually treated with temozolomide.

### Imaging

Imaging was performed pre-surgically, and 1 to 43 days (mean 5.03 days) separated the MRI scan from surgery. MRI was performed on an Achieva 3T Philips system (*Philips*, *Best*, *Netherlands*) with a standard 8 elements head coil. The MRI protocol consisted of 3D T1-weighted images acquired before and after intravenous contrast (TR/TE 8.4/4ms; voxel size 0.94x0.94x1mm^3^; FOV 240x240mm; 0.15mmol/kg gadobutrol); sagittal T2 TSE (TR/TE 3000/80ms; voxel size 0.43x0.43x4mm^3^; FOV 240x231mm), and axial T2FLAIR (TR/TE/TI 11000/125/2800ms; voxel size 0.45x0.45x4mm^3^; FOV 230x183mm). Conventional MRI sequences were supplemented by DSC perfusion with the following parameters: 2D gradient-echo single-shot; TR/TE: 1200/30ms; voxel size 1.6x1.6x4mm^3^; flip angle 60^°^; 100 dynamic images (124s). In order to minimize T1 effects due to contrast leakage, a pre-loading dose of 0.05mmol/kg was administered prior to DSC perfusion. An intravenous contrast bolus of gadobutrol (0.1mmol/kg at 5ml/s followed by 30ml saline) was given after 15 dynamics.

### MRI data analysis

Post-processing of the perfusion was performed using in-house developed modules run in SPM8 (*Statistical Parametric Mapping*, *Wellcome Trust Centre for NeuroImaging*, *Inst*. *of Neurology*, *University College London*, *UK*) and MatLab (*Mathworks*, *Natick*, *MA*, *USA*).

Perfusion DSC data were analyzed using a parametric approach [16,17]. The arterial input function was determined semi-automatically, with a tracer arrival timing-insensitive method [18] guiding the physician. The additional R1 and R2* effects of contrast agent leakage into the extravascular space were taken into account by fitting a gamma variate-based vascular model to the measured R2* curve via a recently published parametric approach [14], albeit with an altered vascular model taking into account the effect of contrast agent leakage, thereby yielding leakage-corrected maps. Color-coded CBV and CTH maps were calculated by using the approach described in ref. [14]. In short, the probability density function of capillary transit times h(τ) is parameterized by a gamma distribution with the parameters α and β.

$$h(\tau |\alpha ,\beta )=-\frac{dR}{dt}=\tau^{\alpha -1}e^{-\tau /\beta }$$

where R represents the residue function defining the proportion of contrast agent remaining in the vasculature at time t after it was injected. The mean transit time (MTT) is determined by the mean value of the gamma distribution defined by parameters α and β, and becomes MTT = α β. Similarly, the CTH is calculated as the standard deviation of the gamma distribution, and becomes $\text{CTH}=\beta \sqrt{\alpha }$.

Because of the inherent properties of capillary networks, MTT and CTH values are naturally correlated in healthy brain tissue. We expected this correlation to be disturbed in tumors, and generated therefore maps of the CTH/MTT ratio, referred to as coefficient of variation (COV) maps in the following.

Contrast-enhanced T1-weighted images were re-sliced to T2FLAIR. A neuroradiologist (AT, five years of experience) outlined the enhancing tumor part, the peri-focal edema (high signal changes on T2FLAIR with the enhancing tumor subtracted), and the contralateral normal appearing white matter (NAWM). In cases of non-enhancing lesions, only T2FLAIR was used to define the enhancing tumor, which was accordingly identical with the edema region of interest (ROI). Normal vessel structures and necrotic areas were avoided. Enhancing tumor, edema, contralateral NAWM ROIs, contrast-enhanced T1, and T2FLAIR were subsequently co-registered and resliced to the localization of the DSC data. CBV and CTH values were normalized with the individual mean values obtained in contralateral NAWM (termed rCBV and rCTH in the following).

### Statistical analysis

Statistical analysis was performed using RStudio (*Boston*, *MA*, *USA*). Voxel-wise values of rCBV, rCTH, and COV values were extracted from the three regions. Their mean values, and their sensitivity and specificity for discriminating HGG from LGG were determined. Receiver operating characteristic (ROC) curves were constructed to determine the diagnostic performance of rCBV, rCTH, and COV, and logistic regression was done to investigate whether the combination of two or more parameters increased the accuracy to differentiate between grades. The enhancing tumor, the peri-focal edema, and a combined region consisting of the enhancing tumor and the edema were analyzed. The area under the ROC curve (AUC) was used to compare accuracy of single predictors and combinations. The relationship between TTP and the predictors rCBV, rCTH, COV, age, and extent of surgical resection (i.e. complete / subtotal resection or biopsy) was examined by the Cox proportional-hazards regression model. TTP was defined as the time from diagnosis until tumor progression or death. Tumor progression was diagnosed on T2FLAIR and contrast-enhanced T1-weighted images applying the RANO criteria [19]. The same survival analysis was done for OS, where OS represents the time from diagnosis to death. Finally, we constructed Kaplan-Meier survival curves for high and low values of rCBV, rCTH, and COV. High values were defined as lying above, low values as lying below the median of the respective parameter. The survival curves of the patients with high and low values were compared by log-rank tests with p≦0.05 defining statistical significance.

## Results

**Fig 1:**
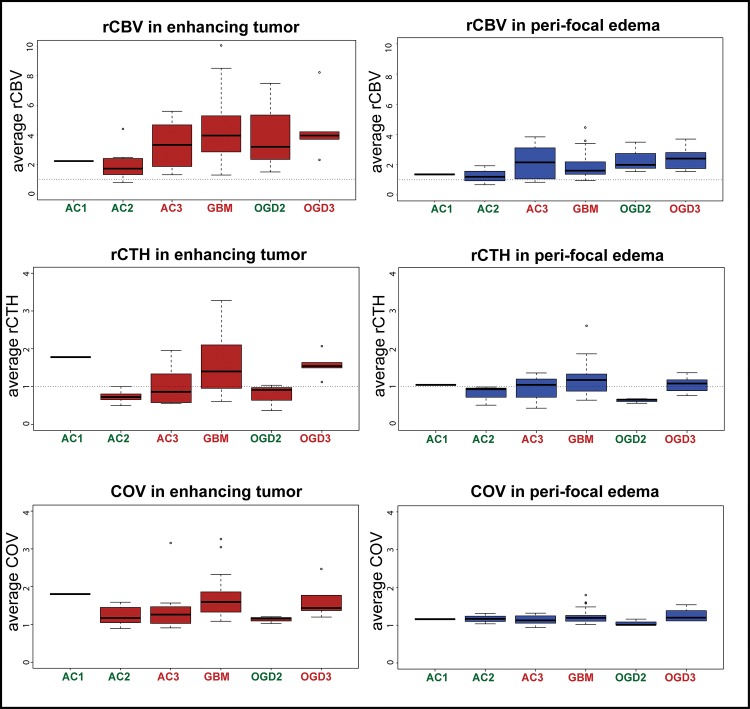
Average values for different tumor types and grades. Boxplots of average rCBV, rCTH, and COV values for different tumor types and grades (bars show median values, boxes represent interquartile ranges). GBM: glioblastoma; AC3: astrocytoma grade 3; AC2+AC1: astrocytoma greade 2 and 1, respectively; ODG3: oligodendroglioma grade 3; ODG2: oligodendroglioma grade 2; HGG: high grade glioma; LGG: low grade glioma.

**Fig 2:**
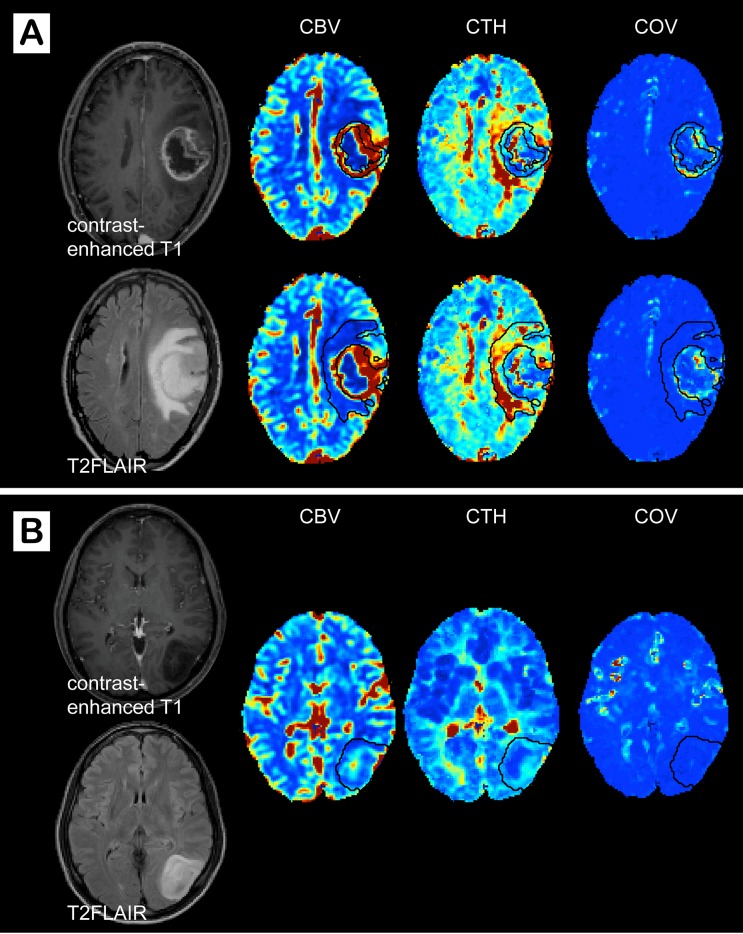
Image examples of a high-grade and a low-grade glioma. Contrast-enhanced T1, T2FLAIR, cerebral blood volume (CBV), capillary transit time heterogeneity (CTH), and coefficient of variance (COV) maps in a patient with (A) a glioblastoma and (B) an astrocytoma grade 2. The enhancing tumor and the peri-focal edema are outlined with black lines on the parameter maps. (A) The glioblastoma shows increased COV in the enhancing part and high CTH in the peri-tumoral edema, whereas (B) the astrocytoma grade 2 is characterized by low CBV, CTH, and COV.

**Fig 3:**
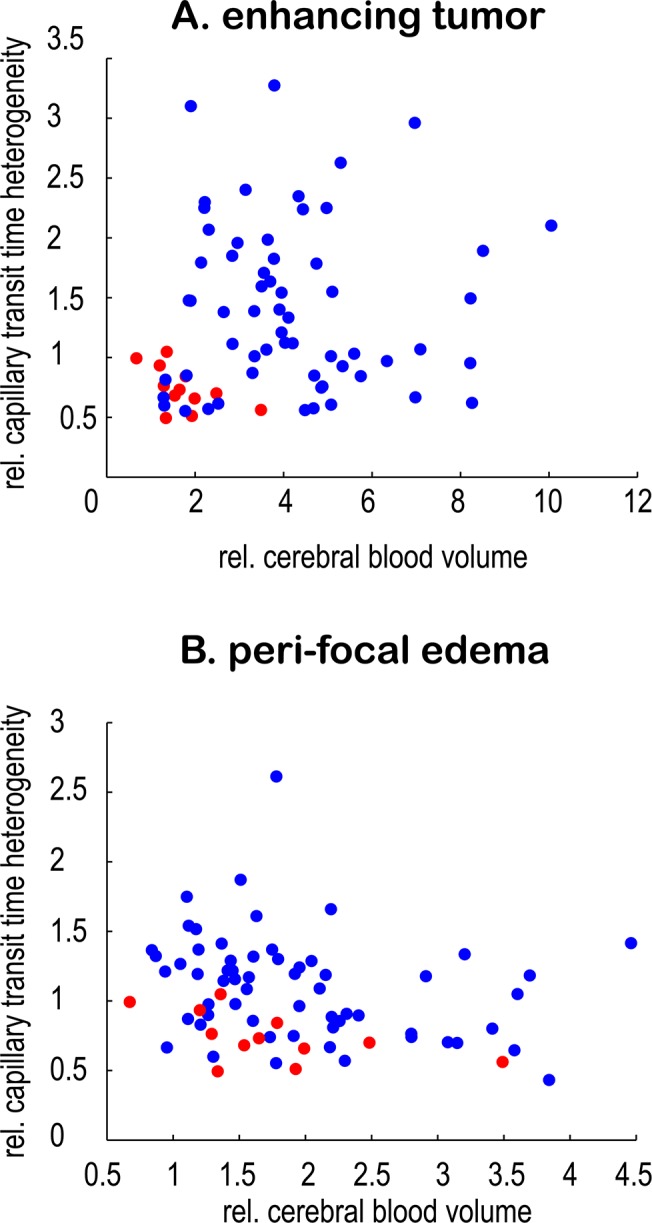
Correlation of rCBV and rCTH. Scatterplots showing the very weak correlation of rCBV and rCTH in (A) the enhancing tumor (R^2^ = 0.01; p = 0.41) and (B) the peri-focal edema (R^2^ = 0.06; p = 0.05), suggesting that they provide different information. Mean values for each patient are plotted. High-grade gliomas are marked with blue, low-grade gliomas with red.

Average values for rCBV, rCTH, and COV for different glioma types and grades are summarized in Table 1. The data distribution is shown as boxplots in Fig 1, and representative image examples are presented in Fig 2. Values of rCBV in the enhancing part of HGG and ODG2 overlap considerably, whereas low-grade astrocytomas have lower rCBV values on average. The same tendency is observed for the peri-focal edema. rCTH is elevated in GBMs and ODG3, but approximately normal or slightly reduced in AC3, AC2, and ODG2. The single AC1 in our study shows relatively high rCTH. COV, as a measure for elevated rCTH in relation to rMTT, is higher in GBM and ODG3, although this difference is less pronounced than the corresponding difference in rCTH. rCTH and COV in peri-focal edema show no differences between groups, except peri-tumoral rCTH in GBM, which tends to be elevated. Moreover, GBMs reveal high COV in the enhancing tumor compared to peri-focal edema (Fig 2A). Fig 3 illustrates that the correlation between rCBV and rCTH is very low (R^2^ in the enhancing tumor: 0.01 (p = 0.41); in the peri-focal edema: 0.06 (p = 0.05)), suggesting that the variables contribute with largely independent information.

**Table 1:** Average values for different glioma types and grades.

	region	rCBV [±SD]	rCTH [±SD]	COV [±SD]
**GBM**	enhancing tumor	4.43(±2.06)	1.55(±0.74)	1.68(±0.50)
	edema	1.86(±0.77)	1.18(±0.83)	1.22(±0.16)
	enhancing tumor & edema	2.74(±1.27)	1.31(±0.46)	1.37(0.27)
**AC3**	enhancing tumor	3.35(±1.46)	0.98(±0.44)	1.37(±0.55)
	edema	2.15(±1.13)	0.97(±0.31)	1.14(±0.12)
	enhancing tumor & edema	2.27(±0.92)	0.92(±0.36)	1.20(±0.20)
**AC2&AC1**	enhancing tumor	2.03(±1.05)	0.85(±0.38)	1.29(±0.30)
	edema	1.29(±0.52)	0.87(±0.25)	1.17(±0.11)
	enhancing tumor & edema	2.17(±0.96)	0.86(±0.32)	1.19(±0.18)
**ODG3**	enhancing tumor	4.48(±2.22)	1.57(±0.34)	1.65(±0.50)
	edema	2.44(±0.86)	1.06(±0.24)	1.27(±0.19)
	enhancing tumor & edema	3.14(±1.60)	1.18(±0.21)	1.36(±0.27)
**ODG2**	enhancing tumor	4.06(±3.09)	0.77(±0.36)	1.13(±0.09)
	edema	2.34(±1.02)	0.63(±0.06)	1.06(±0.09)
	enhancing tumor & edema	2.35(±1.04)	0.63(±0.07)	1.06(±0.09)
**HGG**	enhancing tumor	4.18(±1.97)	1.42(±0.69)	1.61(±0.52)
	edema	1.97(±0.86)	1.13(±0.37)	1.21(±0.16)
	enhancing tumor & edema	2.53(±1.20)	1.13(±0.45)	1.31(±0.25)
**LGG**	enhancing tumor	2.54(±1.84)	0.83(±0.36)	1.25(±0.27)
	edema	1.73(±0.89)	0.80(±0.22)	1.12(±0.11)
	enhancing tumor & edema	1.85(±0.70)	0.77(±0.25)	1.18(±0.17)

Average (± standard deviation; SD) values for different tumor types and grades (GBM: glioblastoma; AC3: astrocytoma grade 3; AC2+AC1: astrocytoma grade 2 and 1, respectively; ODG3: oligodendroglioma grade 3; ODG2: oligodendroglioma grade 2; HGG: high grade glioma; LGG: low grade glioma).

### Differentiation of HGG from LGG

**Fig 4:**
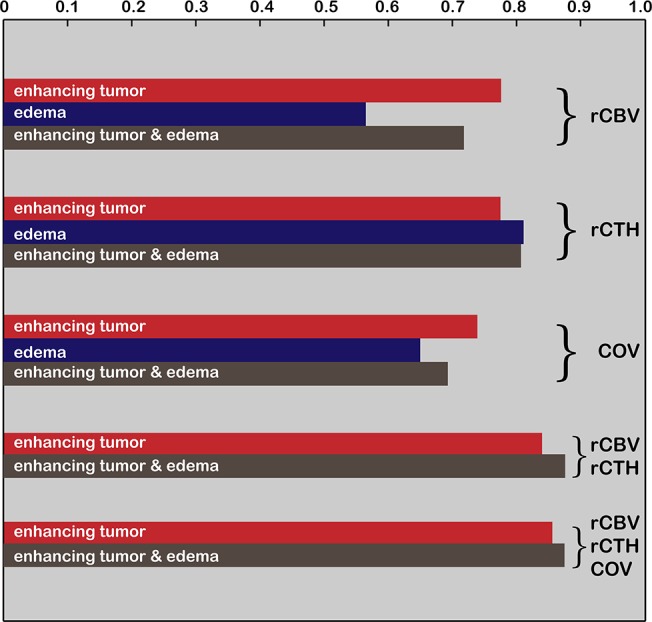
Accuracy for differentiating high-grade from low-grade gliomas. The area under the curve as a measure for diagnostic accuracy is represented by bar length. Accuracy is calculated for single parameters (rCBV, rCTH, and COV) as well as for combinations of predictors, measured in the enhancing tumor (in red), the peri-focal edema (in blue), and a combined enhancing tumor/edema region (in brown).

**Fig 5:**
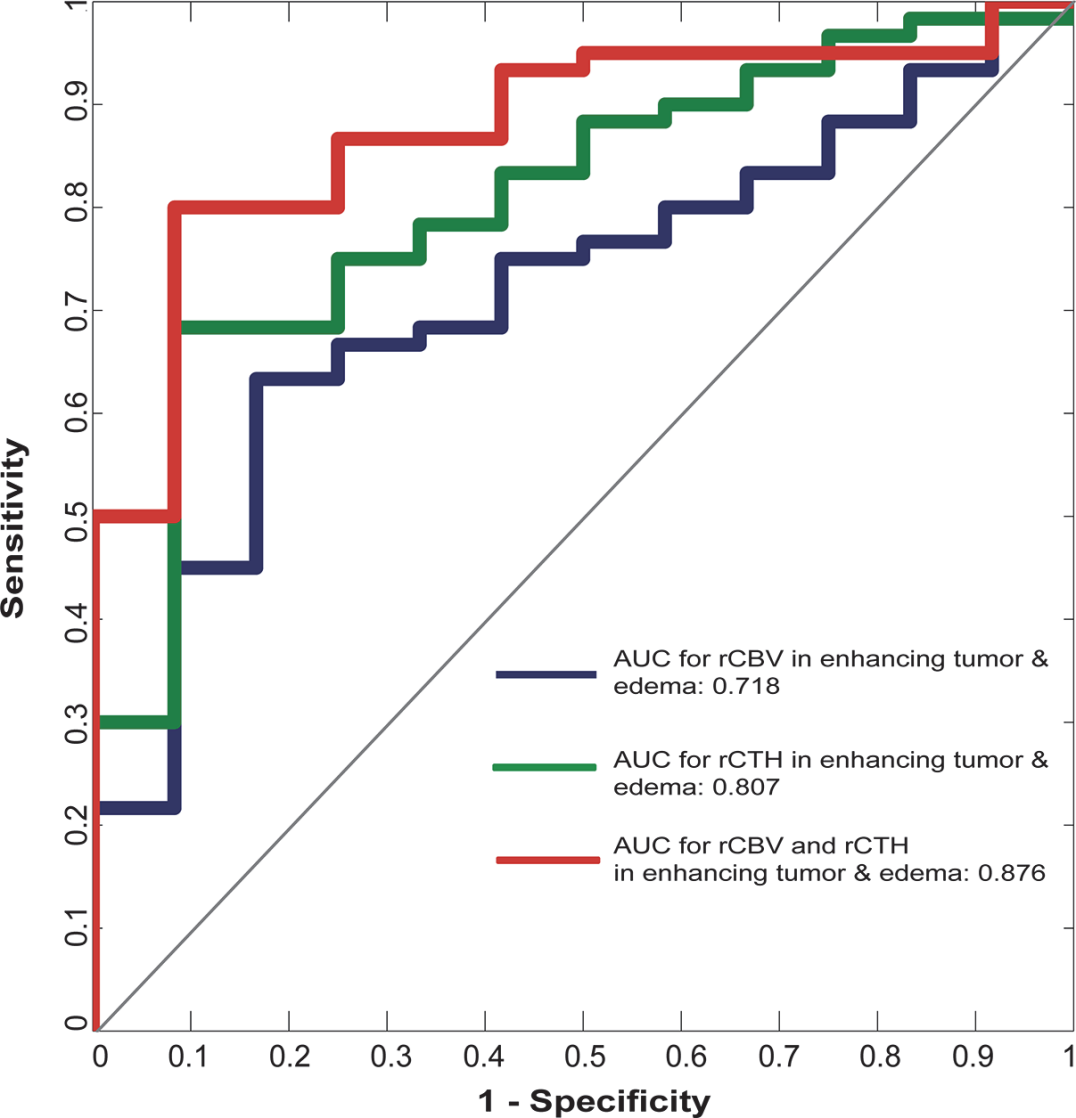
Differentiation of high-grade from low-grade gliomas. Receiver Operating Characteristic curves illustrating the diagnostic performance of rCBV (blue), rCTH (green), and combined rCBV / rCTH (red) mean values within the enhancing tumor and the peri-focal edema to discriminate high-grade from low-grade gliomas.

The AUCs, calculated from ROC curves, to differentiate HGG from LGG are summarized in Table 2 and Fig 4: The highest AUC for single predictors is found for rCTH in the peri-focal edema (AUC = 0.811; p = 0.003), followed by rCTH in a combined region consisting of the enhancing tumor and the peri-focal edema (AUC = 0.807; p = 0.006). rCBV in the enhancing tumor yields 0.776 (p = 0.015). This high accuracy can be slightly improved by combining predictors—see Table 2 and Fig 4. By combining rCBV and rCTH in the enhancing part and edema, an AUC of 0.876 (p = 0.027 for rCBV and 0.006 for rCTH) is achieved, as illustrated by the ROC curve in Fig 5, where the ROC curve for rCBV and rCTH in the enhancing tumor and edema (red) is compared to that of the single predictors rCBV (blue) and rCTH (green), respectively. Moreover, rCTH in the enhancing tumor and edema outperforms rCBV and COV when identifying GBM cases (AUC = 0.763; p<0.001 for rCTH; AUC = 0.700; p = 0.017 for COV; AUC = 0.637; p = 0.09 for rCBV).

**Table 2:** Results of the logistic regression differentiating high-grade from low-grade gliomas.

		AUC HGG/LGG [a.u.]	p HGG/LGG
**rCBV**	enhancing tumor	0.776	*0*.015*
	edema	0.565	0.376
	enhancing tumor & edema	0.718	*0*.034*
**rCTH**	enhancing tumor	0.775	*0*.014*
	edema	0.811	*0*.003*
	enhancing tumor & edema	0.807	*0*.006*
**COV**	enhancing tumor	0.739	*0*.029*
	edema	0.650	0.219
	enhancing tumor & edema	0.693	0.070
**rCBV & rCTH**	enhancing tumor	0.840	*0*.028* (rCBV), *0*.023* (rCTH)
**rCBV & rCTH**	enhancing tumor & edema	0.876	*0*.027* (rCBV), *0*.006* (rCTH)
**rCBV & rCTH & COV**	enhancing tumor	0.856	0.076 (rCBV), *0*.038* (rCTH), 0.437 (COV)
**rCBV & rCTH & COV**	enhancing tumor & edema	0.875	*0*.032* (rCBV), *0*.010* (rCTH), 0.358 (COV)

The area under the curve (AUC) as an accuracy measure for discrimination between high-grade and low-grade gliomas. The AUC is calculated for the three parameters and their combinations in the enhancing tumor, the peri-focal edema, and a combined region of enhancing tumor & peri-focal edema. A p-value ≦ 0.05 (marked with *) allows the conclusion that the predictor has enough strength to distinguish between the two groups.

### Survival analysis

The effect of rCBV, rCTH, COV, age, and the extent of tumor resection on TTP in a combined model shows that only age and rCTH are significantly associated with TTP (p_enh.tumor_ = 0.03; p_enh.tumor&edema_ = 0.02; p_age_<0.001), whereas rCBV also becomes significant when correlating to OS (p_enh.tumor_ = 0.004 for rCBV; p_enh.tumor_ = 0.011 for rCTH; p_enh.tumor&edema_ = 0.02 for rCBV; p_enh.tumor&edema_<0.001 for rCTH; p_age_<0.001).

**Fig 6:**
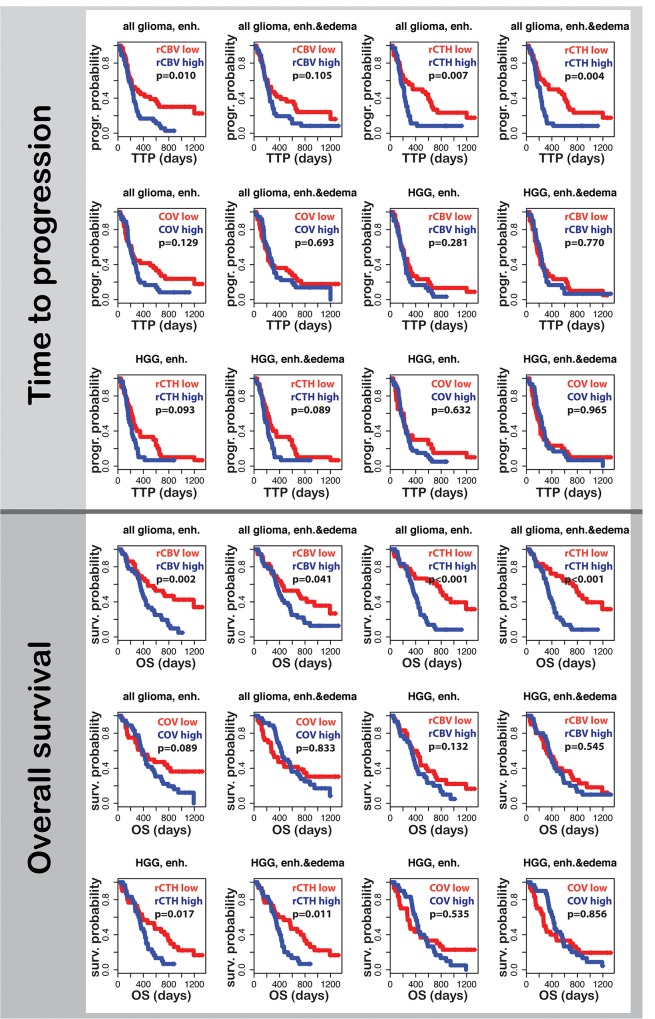
Survival curves for high-grade glioma patients. Time to progression and overall survival for all glioma and for high-grade glioma (HGG) cases are shown. Curves for patients with high mean rCBV, rCTH, and COV values in the enhancing tumor or in a combined region consisting of the enhancing part and the peri-tumoral edema are blue; curves for patients with low mean values are red. A p-value < 0.05 denotes significant difference between the two curves.

Comparing the Kaplan-Meier survival curves, only rCBV and rCTH in the enhancing tumor and rCTH in a combined region consisting of enhancing tumor and edema are associated with TTP and OS, where predicting OS by means of rCTH is highly significant (p < 0.001). Moreover, rCBV in the enhancing part and edema correlates marginally with OS (p = 0.041). When comparing high and low values in the group of patients diagnosed with HGG, the only predictor for OS is rCTH. p-values for all comparisons are reported in Fig 6.

## Discussion

This study confirms that the extent of tumor neovascularization (CBV) is an important diagnostic and prognostic entity in brain tumors and extends this finding by showing that microhemodynamics (CTH) in and around the tumor can be measured as part of a standard MRI protocol. Our data show that knowledge of CTH increases the diagnostic power of CBV in terms of discriminating between HGGs and LGGs, and assists in the identification of GBM. Most importantly, we show that the ability of CTH to predict disease progression or OS outperforms that of the traditional angiogenesis measure, CBV.

### CTH as an imaging biomarker for microvascular changes in tumor angiogenesis

In spite of abundant angiogenesis, as evidenced by the high CBV, most high-grade tumors are found to be under-supplied with oxygen [20]. This has been ascribed to the chaotic architecture of the neo-vessels with numerous arteriolo-venular shunts and compromised capillary paths, which are speculated to lead to hypoxia and eventually necrosis [10,21]. We have recently described how elevated CTH reduces the availability of oxygen, possibly explaining the apparent contradiction between the abundant tumor vascularization, and their hypoxic status [11,13]. As expected, this study showed CTH to be increased in regions with ample angiogenesis, typically in the enhancing part of HGGs. We found that the inclusion of CBV values obtained from voxels within peri-tumoral edema *reduced* the accuracy of diagnostic and prognostic models, whereas it *increased* when including CTH values from peritumoral edema. Elevated CTH in edema might represent the hemodynamic effects of capillary compression, but it might also capture early changes in capillary morphology as a result of hypoxia-induced angiogenesis. The extended flow-diffusion equation shows that control of blood flow through individual capillaries is crucial, in that it prevents blood from passing though the capillary bed at transit times that are too short to permit efficient extraction of oxygen, nutrients, and other solutes. The extent of ‘shunting’ is determined both by blood flow (high blood flow reduces oxygen extraction efficacy across all transit times) and by CTH, which determines the proportion of the blood flow that passes through capillaries at the most extreme transit times. The combination of high CTH and high blood flow is therefore predicted to result in insufficient oxygen extraction, hypoxia, and necrosis [11]. This prediction is supported by findings of Jensen *et al*., who demonstrated an increased expression of hypoxia markers such as hypoxia inducible factor 1 and vascular endothelial growth factor in tissue regions with altered capillary transit times [22,23].

### The role of tumor hypoxia

It is well established that disease progression or death are correlated with the extent of hypoxia in tumor tissue [20,24,25]. This association may be explained by the fact that tumor-initiating stem cells thrive in hypoxic environments. Localized in peri-capillary stem cell niches, tumor stem cells become the origin of proliferating malignant tissue, and the aberrant capillary networks appear to offer optimal conditions for tumor maintenance, as well as therapy resistance [26,27]. While CBV quantifies the extent of neo-angiogenesis, we propose that CTH provides an index of microvascular function in terms of the local extraction of oxygen, glucose and other solutes. CTH may therefore provide information on local tumor microenvironment in relation to the peri-capillary niches in which tumor stem cells reside. Our results indicate that CTH adds diagnostic and predictive information particularly in areas, where angiogenesis has not yet developed to an extent that can be detected by significant CBV increases. We speculate that this prognostic value may be related to the technique’s ability to capture early microenvironmental changes that are highly conducive to tumor growth in malignant tumors.

### CTH as a potential biomarker to monitor anti-angiogenic therapy

There is a growing need to develop sensitive biomarkers to predict tumor responses to anti-angiogenic therapy [28]. Anti-angiogenic therapies were originally developed to inhibit tumor growth by starving them of their nutrient supply [29,30], but subsequent studies have shown that this only happens in some tumors, while in others, tumor oxygenation is improved [31]. The latter observation has been explained by *vascular normalization*, resulting in better oxygenation and higher sensitivity to subsequent radio-chemotherapy [28,32]. Quarles and Schmainda [33] reported transit time normalization in response to two different doses of an anti-angiogenic drug in a murine gliosarcoma model, and our model predicts that the changes in MTT and CTH they reported were consistent with improved oxygenation at one dose, but more severe hypoxia at the other [13]. We speculate that dose- and treatment-related changes in CTH and MTT as a physiological surrogate of vascular normalization can be utilized as a means of optimizing anti-angiogenic therapy in individual patients.

### COV as marker for lost vascular control

We introduced an additional parameter, COV, which measures CTH divided by MTT. In a normal capillary bed, CTH tends to increase linearly with MTT [11], and maps of COV therefore show little contrast in normal tissue (see Fig 2). If CTH is elevated and normal control of blood flow is lost, however, these parameters may be uncoupled, and tissue oxygenation be severely affected. In particular, ‘malignant CTH’, a combination of elevated CTH *and* high flow, has been predicted to lead to a paradox state of severe tissue hypoxia and necrosis [11]. This phenomenon is associated with high COV, which we found in the enhancing tumor and the vicinity of necrosis in many GBMs in our study. While elevated COV may be a marker of metabolic derangement and imminent necrosis, it appears that maps of CTH and CBV are sensitive to a broader spectrum of tumor and peri-focal changes in the diagnosis of brain tumors. COV might, however, be a valuable tool for assessing improvements in tissue oxygenation in individual patients during anti-angiogenic treatment, where normalization of COV normalization would be indicative of a beneficial oxygenation response.

### Limitations

There are several limitations of our study: Although we included a similar number of patients as in comparable studies [34–36], only 17% of our patients showed LGG histopathology, thus limiting our statistical power when evaluating LGG subtype properties. The distribution of patients across glioma types and grades in our study is representative of the general incidence [37], and statistical power could therefore only have been improved by increasing the total patient number, or by a pre-selection of LGGs. Moreover, due to tumor heterogeneity, tissue sampling for histological grading may have missed the most malignant tumor parts in cases of biopsies and subtotal resection. Molecular markers as methylated O^6^-methylguanine-DNA-methyltransferase (MGMT) and possible mutation in the isocitrate-dehydrogenase (IDH) gene were only available in a subset of patients. It was therefore not possible to correct survival analyses for these covariates, which would have added important predictors and should be done in future studies. We included both astrocytoma and oligodendroglioma, which is representative for an unselected, pre-surgical dataset in the clinics, but which introduces potentially a bias into our results. Oligodendroglioma are known to confound the reliability of rCBV differentiating grades owing to their fine capillary network even in low-grade tumors. Excluding them from the analysis increased accordingly the grading accuracy of rCBV in our study, whereas that of rCTH was either unchanged or decreased slightly. The results of our survival analysis were not affected by the inclusion of oligodendroglioma.

The accuracy of perfusion parameters estimated by DSC MRI is inherently affected by disruptions of the blood-brain barrier. The leakage of contrast agent that occurs in most HGGs introduces signal changes that disturb the kinetic analysis of the concentration-time in individual image voxels, and may lead to either over- or underestimation of the calculated parameters, depending on the resulting T1 or T2* effects [38]. This error source can be minimized by the administration of a pre-loading dose of contrast agent and / or by using mathematical corrections during post-processing [39]. We applied both methods, aiming to reduce these sources of error as much as possible.

### CTH as an indirect marker of maximum oxygen availability and potential comparison with other quantitative methods

In this manuscript, we used DSC MRI and tracer kinetic modeling to determine estimates of CTH and MTT, which, in turn, provides an upper limit of tissue oxygenation for a certain tissue oxygen tension [11]. Rather than ‘true’ oxygen extraction, our approach therefore determines the extent to which the tumor microcirculation limits tumor oxygenation—with tissue oxygen tension as an ‘unknown’ in a given patient. CTH is therefore an indirect marker of maximum oxygen availability and not a direct measure for tissue oxygenation. In spite of the different approach and informative value, it would be useful to compare our method to established techniques estimating tissue oxygenation, such as ^18^F-fluoromisonidazole (FMISO) Positron Emission Tomography (PET) [20,24] or near-infrared spectroscopy (NIRS) [40]. Other MRI methods as blood oxygen level dependent (BOLD) contrast MRI have been shown to yield reliable noninvasive estimates of blood oxygen saturation in glial tumors [41,42]. We note that tissue oxygen tension cannot be estimated from blood oxygen levels because of the tendency of tumor vasculature to ‘shunt’ oxygenated blood, but BOLD-based estimates of oxygen saturation together with information on the microvasculature might shed further light onto the development of tumor hypoxia. Emblem et al. have described a promising new vessel architectural imaging approach that captures plasma retention in microvessels, while being sensitive to both their size distribution and blood oxygenation [28]. We suggest that important insights into tumor hypoxia and its impact on patient prognosis can be gained by combining these noninvasive methods.

### Conclusions

In conclusion, our results indicate that CTH adds valuable information on tumor microenvironment in human glioma. We speculate that this information will prove useful for pre-surgical imaging, both regarding grading, expected TTP, and OS. CTH estimates hemodynamic properties of neo-angiogenesis, and most importantly, according to our model, the efficacy of maximum oxygen extraction. CTH might serve as an indirect marker for oxygen availability in tissue and can be calculated from clinically available perfusion-weighted MRI [14]. Moreover, we propose that CTH mapping can improve our understanding of the mechanisms of anti-angiogenic therapy and serve as a potential tool to monitor treatment.
